# Current Update of Collagen Nanomaterials—Fabrication, Characterisation and Its Applications: A Review

**DOI:** 10.3390/pharmaceutics13030316

**Published:** 2021-02-28

**Authors:** Samantha Lo, Mh Busra Fauzi

**Affiliations:** Centre for Tissue Engineering Centre and Regenerative Medicine, Faculty of Medicine, Universiti Kebangsaan Malaysia, Cheras, Kuala Lumpur 56000, Malaysia; P109571@siswa.ukm.edu.my

**Keywords:** nano collagen, tissue engineering, tissue regeneration, three-dimensional, biomaterial, extracellular matrix

## Abstract

Tissue engineering technology is a promising alternative approach for improvement in health management. Biomaterials play a major role, acting as a provisional bioscaffold for tissue repair and regeneration. Collagen a widely studied natural component largely present in the extracellular matrix (ECM) of the human body. It provides mechanical stability with suitable elasticity and strength to various tissues, including skin, bone, tendon, cornea and others. Even though exogenous collagen is commonly used in bioscaffolds, largely in the medical and pharmaceutical fields, nano collagen is a relatively new material involved in nanotechnology with a plethora of unexplored potential. Nano collagen is a form of collagen reduced to a nanoparticulate size, which has its advantages over the common three-dimensional (3D) collagen design, primarily due to its nano-size contributing to a higher surface area-to-volume ratio, aiding in withstanding large loads with minimal tension. It can be produced through different approaches including the electrospinning technique to produce nano collagen fibres resembling natural ECM. Nano collagen can be applied in various medical fields involving bioscaffold insertion or fillers for wound healing improvement; skin, bone, vascular grafting, nerve tissue and articular cartilage regeneration as well as aiding in drug delivery and incorporation for cosmetic purposes.

## 1. Introduction

As of today, many biomaterial research and technological advancements are being made as the demand in cosmeceutical, pharmaceutical and medical applications for biomaterials has tremendously increased [1,2,3,4]. There are many polymers found in nature, such as chitosan, keratin, gelatin, cellulose, elastin and collagen [1,5,6]. Collagen, for example, can be used in products such as leather and parchment along with numerous biopolymeric materials for medical-based products including prosthetics, surgery and wound healing applications [1,7].

**Figure 1:**
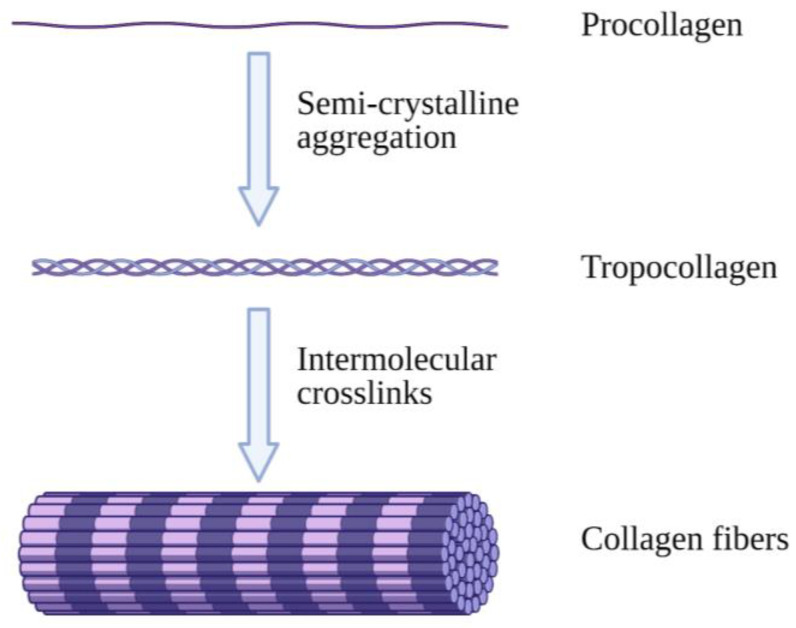
Illustrative image of collagen fibre formation. Collagen molecules intertwine with each other, forming collagen fibrils, which are then combined and form collagen fibres. Created with Biorender.com (Accessed date: 10 February 2021).

Collagen is an abundantly available protein structure in the extracellular matrix (ECM) that is responsible for three-dimensional microstructure sustainability. It is ultimately found in connective tissues of the human body such as skin, tendons, bones and ligaments [8]. There are a total of 28 different collagen types, of which type I is the most commonly found in the body [9]. Type I collagen (Col-I) monomers form triple-helical collagen structures through intertwining to form collagen fibrils, which are then assembled in bundles known as collagen fibres (Figure 1) [10]. The molecular structure of collagen consists of three individual polypeptide chains intertwined together through hydrogen bonds at the CO and NH groups as well as electrostatic interactions. The individual strands of procollagen bond and form collagen fibrils, which contributes to collagen’s stability and flexible characteristics with high mechanical strength [11].

Collagen provides mechanical support in many tissues of mammals and is, hence, present in large quantities in the body. It provides tensile strength in the skin and other organs through its widespread molecular line formation. Fully developed collagen can also combine with hydroxyapatites, which are mineral crystals and typically found in bones and teeth [1]. With all the aforementioned aspects of collagen, progressively many scientists have been creating biomaterials using collagen as a main component since the 1970s [12]. A biomaterial is defined as a material intended for use on biological systems, acting as a medical device [13]. Biomaterials often use polymers of synthetic, natural or a combination of both sources, fabricated to aid or replace biological functions or damaged body parts [14].

Collagen is an ideal component for biomaterial fabrication as, upon application to injury sites on the human body, it is easily degradable through extracellular collagenases, which then aids in tissue regeneration through the resorption of collagen [15]. Collagen substrates also play a role in cell adhesion and migration through cell–collagen interactions in extracellular attachments between collagen and glycoproteins, hence inducing cellular differentiation [16]. The process of wound healing is also sped up through collagen chemotactic enhancement on cells.

Besides that, collagen has high versatility as a biomaterial due to its capability of forming solids or gels through cross-linking. Cross-linking collagen can also reduce the antigenicity of its biomaterial. Collagen is a good material source for biomaterial production because of its low antigenicity with excellent cell-binding properties and biocompatibility.

A certain issue faced with the use of collagen in biomaterials is the presence of telopeptides which have antigenic properties, thus inducing an immune response in the body [17]. However, the antigenic determinants can be removed using pepsin treatment [16]. One study reports that collagen in the quaternary structure such as polymeric collagen is preferred over its monomeric variant due to its ability in initiating thrombus formation through platelet aggregation [18].

## 2. Nano Collagen Fabrication

Nanotechnology is defined as any technological advancement in nano-scaled base materials which can be applied to daily life [19]. It involves the manipulation of matter with at least one dimension with around 1–100 nm in size [19]. On the other hand, nanofibres are defined as cylindrical structures with an external diameter less than 1000 nm, with a ratio of more than 50 between length and width [20]. Nanotechnology utilises matter on atomic, molecular and supramolecular scales for industrial purposes [21]. This technology includes the production of nanomaterials which can be used in different physical, chemical and biological systems, which can be integrated at miniscule levels and larger systems [19].

Nano collagen is regular collagen reduced to the nanometre size. It can be an ideal 3D biomaterial due to its nanoscale-based technology between 1 and 100 nm, providing a high surface area-to-volume ratio, allowing for efficient penetration into wound sites and effective communication [22]. One research paper studied nano collagen fibre-reinforced (NCFR) samples that allow for an evenly distributed fibre formation and organisation. Freeze-dried collagen, in particular, is able to create NCFR samples which are resistant to breakage with elastic properties, making it useful for tissue engineering. The density and the sheer size of nano collagen fibres increases NCFR samples’ matrix interfacial area, enabling it to withstand 500 mN tensile loads with 50 nN load resolution. The crimped characteristic present in nano collagen fibres also adds to their resilience, further enhancing the load transfer capabilities [23]. In this section, different methods have been described for preparing nano collagen 3D structures for various applications.

### 2.1. Electrospinning

**Figure 2:**
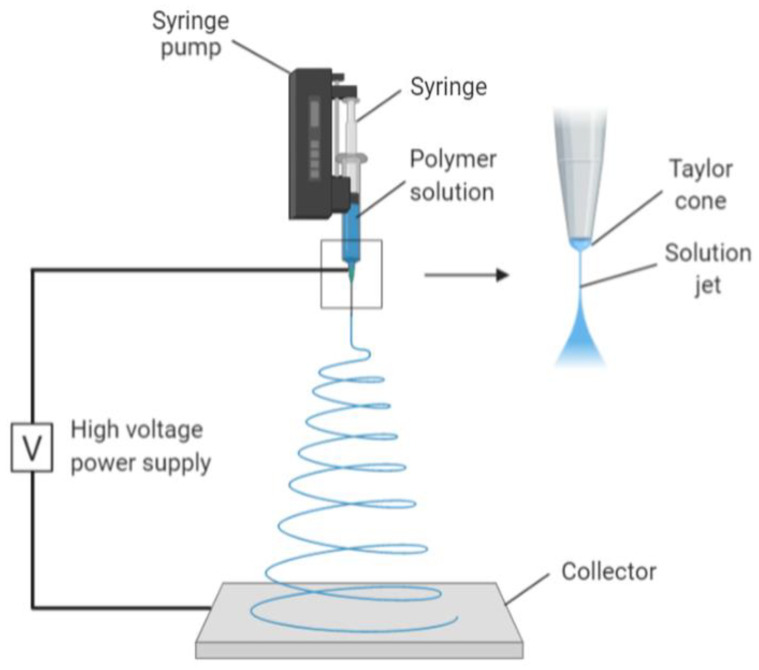
Image mechanism of electrospinning. Collagen polymer solution is fed through the syringe, forming a Taylor cone due to high voltage and low current. The electrostatic forces together with Columbic forces stretch and dehydrate the polymer ejected, forming dry and thin fibres on the collector. Created with Biorender.com (Accessed date: 10 February 2021).

One of the techniques used to create nano collagen is electrospinning. It is a process whereby an electrostatic field is used to form nanofibres from polymeric solutions. It is a commonly used method in creating nanofibres due to its affordability and versatility in producing scaffolds for tissue engineering, creating nanofibrillar matrixes resembling the native ECM. The resulting scaffold has a high surface area-to-volume ratio and a high porosity, both of which are ideal scaffolds for cellular growth and mechanical support [24]. The mechanism of electrospinning involves a spinneret charged with high voltage and low current, followed by the addition of a droplet of polymeric solution (Figure 2). This causes its surface to become highly charged, thus lengthening it to a conical shape, also known as Taylor cone. The shape is due to electrostatic repulsion from the charged droplet surface and Columbic forces from the spinneret. When the electric field reaches a threshold, this electrostatic force surpasses the surface tension and elastic forces of the Taylor cone, thus stretching and whipping it. The complete mechanism removes all solvent molecules, leaving thin, dry fibres in an interconnected and random fashion on a grounded metallic piece. The organisation of the nanofibres during collection is determined by the metallic collector shape (Figure 2) [25].

### 2.2. Nanoemulsion

Collagen can also be fabricated and incorporated through nanoemulsion. A nanoemulsion solution is made from the combination of two immiscible liquids. It can be classified into two phases: oil-in-water (O/W), whereby oil is in the dispersive phase and water in continuous phase. The situation is reversed in water-in-oil (W/O) morphology. Nanoemulsions differ from regular emulsions as the latter fabricate course droplets of up to 1 µm with similar fabrication techniques [26]. The advantages of nanoemulsion technology are that the sheer nano-size of each droplet allows for the active ingredient to be incorporated for uniform dispersion and penetration into the epidermis [27]. Nanoemulsion preparation can be segregated into low-energy, high-energy or combined methods. Low-energy methods involve emulsion inversion point and phase inversion temperature, whereas high-energy methods include high-pressure homogenisation and ultrasonic emulsification. The combined method, however, involves high-shear stirring followed by the diffusion of nano-emulsifiers through interface boundary [28]. Nanoemulsion technology is often used in cosmeceuticals and drug delivery [29]. Nanoemulsions can be used in the cosmetics industry because of the ability to incorporate cosmetic care active ingredients while enhancing their stability and absorption rate [30,31].

### 2.3. Electrospray Deposition

Electrospray deposition (ESD) is a fabrication process of nano-polymers through spraying [32]. ESD is considered a useful method in preparing solid nanoparticles for pharmaceutical purposes [33]. The production of solid particles through ESD uses gentler conditions compared to other nanoparticle fabrication methods while having a relatively simple sample preparation procedure, as all the steps can be carried out in an acrylic chamber at room temperature. Thus, this fabrication process is suitable for fabricating temperature-sensitive polymers such as collagen. The conductivity and viscosity of the polymer solution also play a role in the polymer product outcome through dilution concentration and the addition of ionic solutions such as sodium chloride solution. In a study performed by Nagarajan U et al. (2014), the researchers demonstrated the use of ESD using collagen type I solution for dispersal from a grounded nozzle into a fine mist. An electric field is created from the grounded nozzle and aluminium deposition target charged with a high negative voltage. The solvent present in the collagen solution would evaporate soon after dispersion, leaving only the solid collagen nanoparticles to be collected at the target [34]. ESD has several advantages in comparison to other fabrication methods as it can reduce molecular aggregation and contamination risks of the product while being cost-efficient [35].

### 2.4. Milling

Nano collagen can also be fabricated through milling. Milling is a process whereby mechanical energy is applied onto a polymeric material to be broken down into fine nanoparticles. Milling is an inexpensive way to reduce particle size on large production scales [36]. Mechanical milling such as the ball milling method consists of utilising milling balls for high-energy mechanical collision in polymer breakdown Due to the mechanical and kinetic energy present within the milling vessel, heat energy is produced [37]. Therefore, the vessel must be cooled to prevent overheating or material degradation. Temperature-sensitive components such as collagen can also be mechanically milled at cryogenic temperatures using liquid nitrogen to prevent denaturation from heat [38].

## 3. Characterisation Approaches

### 3.1. Transmission Electron Microscopy (TEM)

There are several methods in determining the characteristics in newly formed nano collagen/collagen fibrils, one of which is transmission electron microscopy (TEM). TEM is a well-known technique in determining collagen fibril parameters, such as fibril length and diameter. These parameters and characteristics will determine cellular function, as any changes will cause tissue alterations. These alterations can result in improper wound healing such as fibrosis and scarring, together with connective tissue dysfunctions such as Ehlers–Danlos syndrome [39]. TEM aims an electron beam condensed with an electromagnetic condenser lens at a plane and passing through the tissue sample. TEM uses an electron scattering technique on the surface of the tissue sample, with more scattering on denser regions, and the images produced are from the contrast of electron backscatter using a block-face scanning method [40]. Serial-section TEM (system) is also used to determine the 3D organisation of nano collagen matrixes. The ssTEM uses transmission electron microscopy in imaging serially sectioned resin-infused tissue samples, with the milled surface facing the scanning electron beam. It relies on a microtome incorporated into the scanning electron microscope to cut thin slices of the tissue sample [41]. However, the disadvantage of the use of TEM involves the irradiation damage caused by the electron beam projection from the electron microscope. Electron irradiation causes damage to tissue samples at a molecular level through excitation and ionisation [42].

### 3.2. Electron Tomography (ET)

Electron tomography (ET) is a tomographic technique which images 3D structures of biomaterials at nano-sizes using TEM. With this technique, nano collagen fibrils have micrographs taken at different angles and orientation through tilting, all of which are then combined to produce a 3D structure [43]. To image biological structures in nanometres, nanographs of the sample are taken at every tilt degree possible on its axis within range. These images are then projected back to produce a 3D image. However, this back-projection leads to image blurring when assembled onto a 3D reconstruction space [44]. To overcome this issue, visual noise can be cancelled out by increasing higher frequencies with a weighting filter of Fourier space [45].

### 3.3. Scanning Electron Microscopy (SEM)

Scanning electron microscopy (SEM) is a technique used in analysing materials at the nanometre scale. It has a magnification of at least 300,000×, which enables crisp imaging production [46]. In SEM, an electron beam is focused onto the surface of a tissue sample systematically, producing a lot of signals that are reflected off the sample surface and altered into visual signals on a cathode ray tube (CRT). The final image produced is highly dependent on the signals from the electron beam and tissue sample interaction [47]. SEM is used to image nano collagen scaffolds to determine their superficial morphology as well as their composition, crystallography and orientation. The sample preparation for SEM is also relatively easy as there is no emphasis on the sample thickness [48]. A study by Duong et al. (2018) investigating the effects of nano collagen aerogels used SEM to determine nano collagen fibril formation and assembly at different levels [49]. In addition to ssTEM, as previously mentioned, serial block-face SEM [SBF-SEM] can also be used to supplement the images produced with ssTEM as it uses automated serial sectioning of tissue samples with electron microscopy compared to the mechanical microtomy approach in ssTEM. This leads to SBF-SEM having the ability to occasionally replace ssTEM in nano collagen fibril organisation imaging [39].

### 3.4. Focused Ion Beam (FIB) Microscopy

Focused ion beam (FIB) microscopy is a machine analysis method that offers high-resolution microstructure imaging and material micro-cutting, hence making it a highly sought-after appliance for the study of structures in life sciences. FIB microscopy has similar mechanisms to SEM, with the only difference being that FIB uses an ion beam on samples while SEM uses an electron beam. Ion beams have a short wavelength but a high energy potential, and FIB microscopy has the capability to produce structures at micro- and nanoscale. High-resolution images are achieved through the production of secondary electrons from reaction of the ion beam on the samples. Most FIB systems use gallium (Ga) ions as they sputter onto the tissue sample, which aids in accurate micromachining. Ga ions are used in FIB microscopy for their low melting point and vaporising pressure characteristics, as well as having a momentum transfer of 30 keV which is usually found in heavier ions [50]. A more technologically advanced FIB is incorporated with SEM and used as a dual system, though more on FIB-TEM and FIB-SEM will be explained in the following sections [51].

#### 3.4.1. FIB-TEM

The preparation of TEM samples has always been challenging to researchers as it has specific requirements of samples being 200 nm or smaller and must allow electrons to pass through it [52]. This led to the usage of FIBs to prepare TEM samples as they can mill thin layers of various sample types into nanometre thickness [53]. A study completed by Simon et al. (2018) utilised FIB-TEM in examining the nanostructure of an osteocalcin nanocomplex from human and rat bones, successfully imaging its collagen and apatite components at nanoscale [54]. Another study performed by Lacoviello et al. (2020) demonstrates the use of FIB-TEM in determining the nanostructures of collagen fibres and crystallinity of Heloderma suspectum osteoderms [55].

#### 3.4.2. FIB-SEM

FIB-SEM is a micromilling and analysis system that can produce 3D images of a tissue sample for characterising nanostructures and organisation [56]. FIB-SEM allows for serial milling, sectioning and imaging simultaneously [57]. The benefits of using a dual-beamed FIB-SEM system include the milling and preparation of tissue samples which are particularly fragile or porous [58]. The ion source of FIB-SEM can also have an impact on the system’s performance [59]. A study compared two ion sources in a dual-beam FIB-SEM system, namely a xenon plasma ion source (PFIB) and a gallium liquid ion source (GFIB) [59]. Like FIB-TEM, FIB-SEM with GFIB is also often used in tissue sample studies. However, it possesses a limitation of inefficient ion removal [58]. FIB-SEM with PFIB gives a two orders greater milling intensity rate as compared to using GFIB [60]. In a collagen scaffold study conducted by Hu et al. (2018) on collagen-hydroxyapatite (Col-HA) composites, the use of FIB/SEM aided in producing high-quality imaging of the collagen structure with clear detail [59]. This is because FIB-SEM can be used to examine the structure and morphology of collagen scaffolds at nanoscale [56].

### 3.5. Fourier-Transform Infrared (FTIR) Spectroscopy

Fourier-transform infrared (FTIR) spectroscopy is a technique which utilises a mathematical process called Fourier transform to transfer data obtained into an absorption and emission spectrum using an interferogram [61]. The data obtained would be in the form of solid, liquid or gaseous state materials, and its usage is advantageous to study samples as it can measure a wide range of spectra and still capture high-quality data results. FTIR spectroscopy functions by emitting a wide range of light frequencies at a sample, and the amount absorbed by the sample is translated into data read in a computer. These steps are then repeated several times over. FTIR spectroscopy can be applied in many fields; however, for this review, only its application on biological materials will be further explained. The FTIR spectroscopy technique allows for sample composition characterisation through raw data translated onto a result spectrum detected from the various components present. FTIR spectroscopy can be used to analyse a variety of biological samples ranging from DNA to organs [62]. A study also utilised Attenuated Total Reflectance Fourier Transform Infrared (ATR-FTIR) spectroscopy together with SDS-PAGE to identify alpha chains, which are characteristic to electrospun collagen, which gives further understanding of bonds within nanostructures [63]. FTIR resolution can be further increased by combining the spectroscopy onto a scanning near-field optical microscopy (SNOM) platform [64]. This combination of techniques can examine and characterise samples at nanoscale and is, hence, labelled as nano-FTIR [65]. Table 1 shows the advantages and disadvantages of each of the characterisation approach techniques mentioned.

## 4. Applications

### 4.1. Skin Wound Healing

Wound healing follows a specific sequential repair process which includes haemostasis, inflammation, proliferation and remodelling [73]. With these specific phases, disruptions in any one of them can prolong the healing time and may completely heal with poor morphology [22]. Common issues faced in wound healing involve pathogens growing and colonising the skin injury. These pathogenic infections may cause inflammation to the skin and can halt its healing process [74]. To reduce the chances/prevent these complications from occurring, there are several types of wound dressings and dermal substitutes such as Graftskin and Dermagraft currently available on the market [75,76]. However, these treatments have a tendency to leave the user uncomfortable and have a high chance of leaving the wound incompletely healed despite proper usage [77]. Therefore, alternative therapeutic three-dimensional biomaterials are needed for better wound healing as well as physicochemical properties and biocompatibility properties [22].

**Figure 3:**
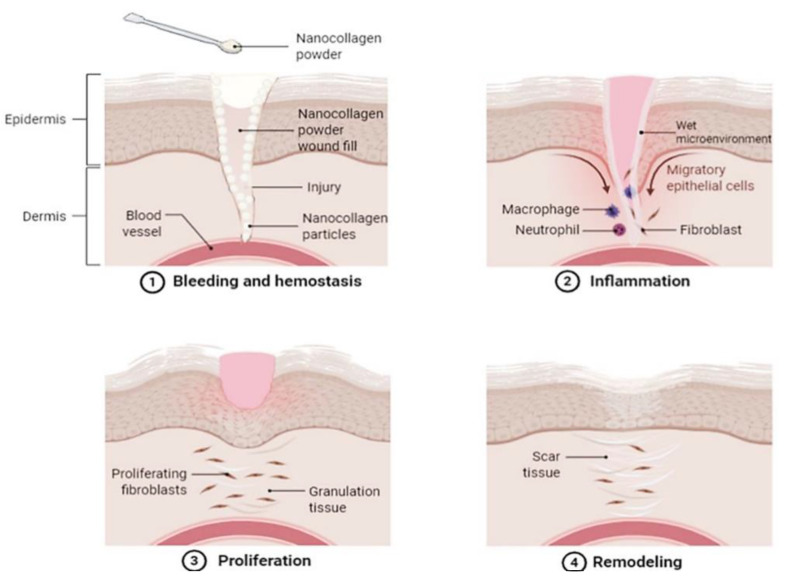
An illustrative image demonstrating a proposed skin wound-healing process with the application of nano collagen powder. The image demonstrates how the powder is applied and fills in the skin wound, where its nano-sized particles better attach to all the crevices of the wound. The nano collagen powder then absorbs exudate secretion and blood, creating a wet microenvironment which acts as a temporary bio-template in the wound. This allows the damaged cells to absorb and benefit from the collagen applied, enhancing healing by encouraging cell migration and proliferation. Created with Biorender.com (Accessed date: 10 February 2021).

An alternative to dermal substitutes can be collagen-based dressings such as powdered collagen or nano collagen powder [78]. Collagen dressings are beneficial for skin wound healing as they are primarily main extracellular matrix and proven with a slow biodegradation rate, hence making them ideal for accelerating wound healing by increasing cell attachment and migration to the wound site [79]. Collagen in the powdered form also has its individual benefits to skin wound healing as powder particles can better attach and coat the fissures of wounds (Figure 3), hence creating an active site for fibronectin binding and fibroblasts growth [78].

Besides collagen powders, collagen nanofibres can also contribute to skin wound healing. As collagen is an extremely pliable protein, it can be fabricated into various forms, one of which is nanofibres. One method through which collagen nanofibres can be fabricated is electrospinning. Electrospinning can result in aligned or random collagen nanofibres, both of which have different purposes in tissue engineering, as the fibre alignment can determine its mechanical properties and cellular activities. Electrospun collagen fibres are considered better compared to other polymer nanofibres due to collagen being present largely in the ECM. The structure of collagen nanofibres also highly resembles the natural structure of native tissues, with low immunogenicity and good biocompatibility [24]. Several studies have shown that the use of collagen nanofibres induces keratinocyte proliferation and differentiation and increases epithelialisation as well as collagen deposition and granulation, all of which are important factors in aiding skin wound healing [25].

In wound healing, the large surface area of the nano collagen scaffold along with its physicochemical properties aid in its systematic cross-talk and mean that it can easily enter the wounded skin, indicating its ability in healing skin wounds as well as delivering regulated amounts of therapeutics [22]. An example of these therapeutics includes the delivery of gold nanoparticles (AuNPs) with wound-healing and tissue-regenerative properties. AuNPs are incorporated into nano collagen scaffolds which can then interact with other components such as growth factors and cell adhesion molecules. They are capable of reducing inflammation as well as encouraging granulation tissue formation and have no rejection issues, making them ideal for wound healing. Another study conducted on rats with cutaneous wounds through applying AuNPs topically revealed re-epithelisation, increased collagen fibre content and granulation tissue formation [80].

Besides AuNPs, wound dressings with silver nanoparticles (AgNPs) are also widely used as they aid in treating deep wounds such as burns and infections [81]. AgNPs have a wide range of antibacterial properties without causing bacterial resistance [82]. AgNPs incorporated into collagen scaffolds make an ideal wound dressing as they have antibacterial properties and can be used for an extended time without being a hindrance to the user [83]. A study demonstrated the AgNPs loaded in an electrospun collagen nanofibre mat displayed antimicrobial activity and anti-inflammatory full-thickness skin wound healing, where an in vivo study demonstrated accelerated wound healing through re-epithelialisation [84]. AgNPs eliminate pathogens in wounds through hindering quorum sensing in bacteria, disrupting biofilm production and removing toxins [85,86].

Natural ingredients from plants such as curcumin (CUR) are known to be antimicrobial with anti-inflammatory properties. The incorporation of CUR with collagen scaffolds displayed wound contraction, complete epithelisation and the formation of granulated tissues [87]. This indicates that CUR-incorporated nano collagen scaffolds can act as a template for skin regeneration. However, plant-based biopolymeric materials for wound treatment are not yet mass-produced as characteristics such as skin reactivity and biodegradability as a wound dressing have yet to be evaluated [88]. Table 2 demonstrates other studies on nano collagen fabrication, intervention and its potential use on skin wounds.

### 4.2. Bone Grafting

Fibrous collagen plays a role in the early stages of bone tissue formation. In the ECM, collagen fibres with diameters of 50–500 nm are placed into bone construction. These collagen fibres then act as a scaffold for the deposition of apatite crystals, continuing bone maturation [91]. The scaffold also aids in cellular formation characteristics such as differentiation and proliferation [92]. Therefore, it is important for bioscaffolds to imitate the natural structure for optimal bone grafting.

Bone tissue engineering technology is ever advancing as bone-related diseases and traumas are hard to treat due to the challenging nature of bone healing and complications with bone loss from non-sterile wounds which lead to infections [93]. To combat this issue, a study performed by Sun et al. (2015) incorporated AgNPs into collagen scaffolds together with bone morphogenetic protein 2 (BMP-2) to aid infected bone defect healing. As mentioned previously, the incorporation of AgNPs into collagen scaffolds allow the controlled release of Ag+, which gives them antibacterial properties. BMP-2 was also included as it greatly induces osteogenesis. Hypothetically, the BMP-2/AgNP/collagen scaffold would be an ideal therapeutic agent as it can heal bone defects while preventing infections. This hypothesis was proven true as the study showed increased expressions of runt-related transcription factor 2 (RUNX2), osteopontin and osteonectin, which increased bone marrow-derived mesenchymal stromal cell (BMSC) differentiation [94]. Other studies have also shown that collagen/chitosan membranes with chitosan nanoparticles support bone regeneration with biocompatibility and physicochemical properties [95].

A study by Wang et al. (2015) was conducted on nano collagen bone production and formation for alveolar ridge preservation. Poor alveolar ridge post-tooth extraction occurs when a repair is not conducted in time or due to poor dental/oral hygiene habits and knowledge. In order to protect the residual ridge post-tooth extraction, artificial nano collagen bone was inserted into patients followed by multi-slice computed tomography (CT) scans to evaluate the alveolar bone mineral density right after implantation. The scan was conducted again 3 months later and it was found that the residual ridge had fused with the nano collagen artificial bone along with a higher alveolar bone mineral density [96]. This indicates that nano collagen artificial bone can be used for alveolar ridge preservation. Another study done by Shen et al. (2015) showed that nano collagen-based bone together with allogenic adipose-derived stem cells can be used to mend ulna bone defects through testing on rabbits, where the results showed an increase in bone mineral density as well as the rate of solid fusion [97].

### 4.3. Drug Delivery

Besides that, collagen nanoparticles may also facilitate in healing processes, as these nanomaterials can also act as therapeutic treatment carriers [22]. New nanotechnology developments are aiming to create collagen scaffolds that are not adversely reactive towards the drug to be delivered to a specific site, while also being released in a controlled manner [98]. Collagen nanoparticles are a prime example of controlled drug-releasing bioscaffolds due to their size and absorptive capabilities [99]. For example, collagen nanoparticles can aid in the infiltration of tumours for the delivery of anti-cancer therapeutics, with the benefit of collagen having a resemblance to the tumour microenvironment [100].

Fabricated gold-loaded hydroxyapatite collagen nano-biomaterials optimised for the drug doxorubicin also displayed wound-healing properties by promoting cellular adhesion, growth and proliferation while having biocompatibility and bioactive properties [101]. A separate study on doxorubicin was also performed for cancer drug delivery. It involved the fabrication of collagen peptide-functionalised chitosan nanoparticles, which resulted in a unique polymeric gel with high encapsulation rates and pH-controlled release [102].

Poloxamer 407 (PM) is a water-soluble triblock polymer which aids in ophthalmic drug delivery. Its incorporation into cellulose nano collagen particles has shown an improvement in PM drug delivery of Ketolorac Tromethamine (KT) through its controlled release in vitro. The nano collagen scaffold decreased the PM critical gelation concentration and strengthened its gel formation, all while extending drug release compared to virgin PM gel. Thus, this nanocomposite combination is an ideal ophthalmic drug delivery method [103].

### 4.4. Nerve Tissue

Nano collagen scaffold grafting has also been used in nerve tissue engineering for the purpose of regeneration. For many years, nerve tissue damage treatment has been highly sought-after as any form of trauma or malfunction imposes serious consequences of mobile disability [104]. Nerve autografts have previously been used for treatments, which, while effective, potentially bring secondary complications such as deformity and donor site shortage. This encourages the development of collagen scaffold nerve tissue implantation which can complete the nerve gaps and produce results equivalent to autografting [105].

Nano collagen scaffolds have been studied and tested for their ability to conform to ECM characteristics as well as aid cellular regeneration [106]. Collagen nanospheres in an injectable form can potentially be administered to release therapeutic drugs/components locally to prevent further neurodegeneration, deliver stem cells or for structural support [107]. Collagen nanofibres were also studied with a longitudinal orientation that displayed the ability to guide cell alignment and neurite outgrowth [106]. The study completed by Zhang et al. (2019) demonstrated the use of collagen/nano-sized β-tricalcium phosphate conduits with the addition of collagen filaments and nerve growth factor for facial nerve regeneration. The study outcomes were improved compound muscle action potential with larger axon diameter and thicker myelin sheath, demonstrating a highly promising study for nerve regeneration [108].

### 4.5. Vascular Grafting

Cardiovascular disease (CVD) causes the most deaths in the world. CVDs such as atherosclerosis and deep vein thrombosis often cause the narrowing or blockage of blood vessel lumen, which can lead to decreased oxygen supply to organs, causing tissue damage. This may lead to the need for surgeries to repair or replace the damaged blood vessel. One such example would be the use of tissue-engineered vascular grafts (TEVGs).

TEVGs utilise modern technology or components to create vascular medical implants [109]. To develop TEVGs, collagen is often used as the scaffold along with the insertion of other components in order to create a sustainable vascular graft. This is because the nano collagen matrix acts as a template for cellular recognition and growth into the vascular graft, leading to successful cell–scaffold incorporation and, hence, a functioning scaffold-based TEVG [109].

A study performed by Park et al. (2019) demonstrated the fabrication of a functional bi-layered structure with poly-ε-caprolactone (PCL) and collagen. The PCL/collagen structure displayed longitudinally aligned nanofibres which aid in cellular adhesion and migration, hence encouraging the re-endothelialisation of a vascular graft [110]. Another similar study used a nanofibrous polycaprolactone bilayer with a collagen coating for vascular tissue engineering. It demonstrates a porous network together with improved mechanical and viscoelastic properties, hence creating a potential scaffold for soft tissue replacement [111].

### 4.6. Articular Cartilage

Articular cartilage is a type of cartilage that functions to reduce friction between articulated bones. One of the characteristics of articular cartilage is that it is avascular in nature, thus lacking the ability to self-heal. Commonly, surgical intervention is required for articular cartilage treatment, such as microfracture (MF), autologous chondrocytes implantation (ACI) and osteochondral transplant (OCT) [112,113]. Due to several limitations of the current available treatments, cartilage tissue engineering (CTE) is on the rise and an advancing field of research [113].

**Figure 4:**
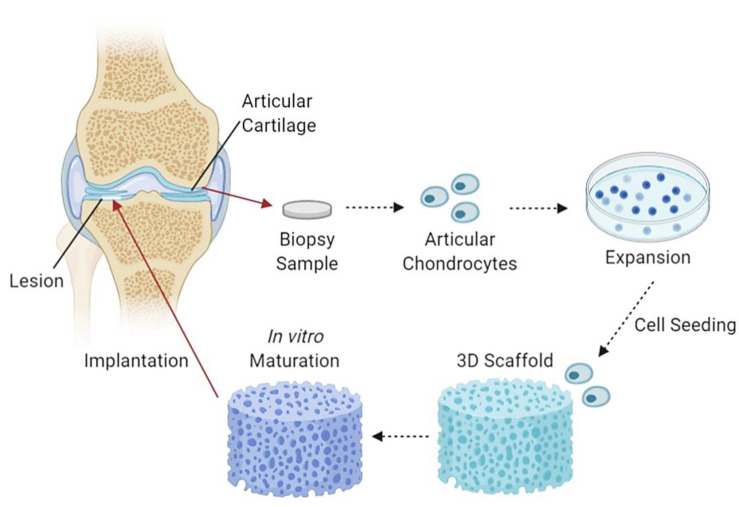
An illustrative flow diagram of cartilage tissue engineering. It begins with taking a biopsy sample of articular cartilage containing articular chondrocytes, which are then cultured, seeded and incorporated into a 3D scaffold, left to mature partially in in vitro conditions and followed by scaffold implantation into chondral lesions [116]. Created with Biorender.com (Accessed date: 10 February 2021).

CTE utilises 3D collagen scaffolds and cells which are initially formed and combined in vitro to partial maturity, followed by implantation into damaged articular cartilages. The production of CTE aims to reduce and eliminate the use of cartilage transplants and other ineffective procedures. An example of a study with promising CTE fabricated a chitosan/collagen/nanohydroxyapatite hydrogel nanocomposite. The results show a highly porous network suitable for efficient convective fluid transport, with thermal stability up to 90 °C and evenly distributed nanohydroxyapatite particles within the chitosan-collagen matrix [114]. Another study demonstrated the use of a nanohydroxyapatite/collagen scaffold that displayed inhibition of chondrocyte dedifferentiation and aided in cellular expansion and growth together with the increment in cartilage-specific ECM factor expression [115]. Figure 4 demonstrates an illustration diagram of cartilage tissue engineering.

### 4.7. Cosmetics

Collagen is a material that has always been a staple in the cosmetics industry and is often used for creating skin care and formulating lip products [117]. It is said that externally applied collagen can encourage collagen production in the dermis; however, this statement does not have sufficient scientific research support [118]. Formulating collagen into beauty products has shown benefits in improving skin health, moisture and elasticity and, together with daily usage, will reduce signs of aging [119]. Collagen in the beauty industry is often sourced from land animals such as bovine and porcine [117]. However, these collagen sources may be a cause of concern regarding religious beliefs, especially in the use of bovine and porcine to Hindus and Muslims, respectively [120]. Using these collagen sources might also be a threat to public health as it can bring on diseases such as bovine spongiform encephalopathy (BSE) and tapeworm infections [117].

A study conducted by Trilaksani et al. (2020) used grouper swim bladder nano collagen as an alternative to land animal collagen to investigate and compare its quality while adhering to cosmetic standards [117]. It was found that the size of the collagen molecules used can play a role in their effectiveness as smaller-sized—hence, nano—collagen has also been shown to produce positive effects in terms of skin health as it has better penetration abilities to treat skin concerns [117]. All in all, nano collagen from grouper swim bladder complied to the collagen standards for cosmetic material creation as well [117]. This indicates that grouper swim bladder nano collagen is just as effective as terrestrial animal collagen, with the benefits of avoiding complications in religious beliefs and diseases.

## 5. Future Perspectives

The future of nanotechnology in collagen is promising as it is a scientific field that is ever-advancing and will continue to do so [121]. Nano collagen has its prospective advantages such as the ability to deliver localised treatment and therapeutic factors while providing a sustainable microenvironment at injury sites to encourage healing and cellular growth [107]. However, various drawbacks are also present in nanotechnology as certain unmet challenges in healing, particularly in the case of skin wounds, have many limitations with low success potential, and only a few therapeutic agents are approved for commercial use [121]. Nanotechnology is also faced with the hindrance of complex pathophysiological expressions with insufficient knowledge in nanoparticle mechanisms of action in the body [122]. Due to the lack of knowledge, nanoparticles may bring on unexpected negative effects such as inflammation and possible toxicity [123]. However, nanotechnology is still an ongoing trend in the scientific research field with a plethora of untapped potential, despite the drawbacks, which aids in the hope that future studies may reduce its disadvantages, fabricating safe and effective nano-based products. Therefore, more research and studies must be conducted in order to produce approved therapeutic agents that reap the benefits of nanotechnology while being biocompatible to the human body [121].

## 6. Conclusions

In conclusion, this review paper describes that nano collagen and collagen scaffolds themselves are very important aspects in clinical settings and can be used in potentially life-saving technology. Collagen is a naturally occurring scaffold found abundantly in the human body, with the role of mechanical support. While engineered collagen scaffolds are common, developing nano collagen scaffolds is a relatively new technology and poses challenges. Due to nano collagen’s small size, it has a large surface area-to-volume ratio and is highly resilient to mechanical stress. Nano collagen can be fabricated using electrospinning technology, which artificially spins nano collagen fibres to mimic ECM and creates a scaffold. Various techniques have been used to characterise fabricated nano collagen, including TEM, ET, SEM, FIB and FTIR, to ensure the quality of the nano-size achieved. Nano collagen scaffolds can be applied in many forms of treatments and enhancements, including wound healing, bone grafting, drug delivery, nerve tissue regeneration, vascular grafting, articular cartilage regeneration and cosmetics. Obviously, nano collagen is an advancing form of nanotechnology; hence, more research should be conducted to further develop this technology in the hopes that, in the future, nano collagen scaffolds will be more accessible to the public.

## Figures and Tables

**Table 1:** The advantages and disadvantages of each characterisation approach technique mentioned.

Characterisation Techniques	Advantages	Disadvantages
Transmission electron microscopy (TEM)	Straightforward sample preparation technique. Unsusceptible to radiation.	Presents structural artifacts from staining and dehydration [66].
Electron tomography (ET)	Produces 3D structural organisation data of sample.	Cannot be tested on live specimens [67]. Produces low-quality reconstructions of data [68].
Scanning electron microscopy (SEM)	Images 3D topographic data of samples.	Can cause structural abnormalities in samples from harsh preparation processes [67].
Focused ion beam (FIB) spectroscopy	2D samples can be imaged at nanoscale.	Ion beam emission causes surface damage on samples [69].
FIB-TEM	Allows site-specific sample preparation.	Sample surface damage from ion implantation [70].
FIB-SEM	Produces detailed surface images of 2D flat milled samples.	Limited sample processing methods. Expensive to conduct [71].
Fourier transform infrared (FTIR) spectroscopy	Fast and sensitive quantitative analysis technique.	Uses only a single beam for quantitative analysis [72].

**Table 2:** Description of other studies that have been conducted on nano collagen fabrication, intervention and its potential use on skin wounds.

Author(s)	Nano Collagen Source	Summary of Intervention	Results
Pringgandini LA et al., 2018 [89]	Goldfish scales *(Cyprinus carpio)*	Collagen extraction and purification followed by freeze-drying, producing nano collagen powder spray.	Accelerated healing of incision wound in mice.
Kochar MP et al., 2020 [90]	Collofiber-MM™, collagen type I, naturally sourced (not specified)	Fabrication process not stated, powder product used consists of collagen nano particles, mupirocin and metronidazole and was applied onto chronic ulcers of 100 patients.	Re-epithelialisation and early ulcer healing, reduces exudate secretion and bacterial colonisation.
Van Duong H et al., 2018 [49]	Catfish skin waste (*Pangasius bocourti*)	Fabrication of water-dispersible nano collagen helices, can be made into sponge-like aerogels.	Highly versatile applications as wound dressings, bandages and tissue-engineering scaffolds.

## Data Availability

Not applicable.
